# Comparison of an in-house real-time duplex PCR assay with commercial HOLOGIC® APTIMA assays for the detection of *Neisseria gonorrhoeae* and *Chlamydia trachomatis* in urine and extra-genital specimens

**DOI:** 10.1186/s12879-018-3629-0

**Published:** 2019-01-03

**Authors:** Johanna M. E. Venter, Precious M. Mahlangu, Etienne E. Müller, David A. Lewis, Kevin Rebe, Helen Struthers, James McIntyre, Ranmini S. Kularatne

**Affiliations:** 1 Centre for HIV and Sexually Transmitted Infections, National Institute for Communicable Diseases (NICD), National Health Laboratory Service (NHLS), Johannesburg, South Africa; 2 Western Sydney Sexual Health Centre, Western Sydney Local Health District, Parramatta, Australia; 3 Marie Bashir Institute for Infectious Diseases and Biosecurity & Sydney Medical School, Westmead, University of Sydney, Sydney, Australia; 4 Anova Health Institute, Johannesburg, Cape Town South Africa; 5 Division of Infectious Diseases and HIV Medicine, Department of Medicine, University of Cape Town, Cape Town, South Africa; 6 Division of Epidemiology & Biostatistics, School of Public & Family Medicine, University of Cape Town, Cape Town, South Africa; 7 Department of Clinical Microbiology & Infectious Diseases, Faculty of Health Sciences, University of the Witwatersrand, Johannesburg, South Africa

**Keywords:** *Neisseria gonorrhoeae*, *Chlamydia trachomatis*, In-house real-time PCR, APTIMA, Urine, Extra-genital

## Abstract

**Background:**

Extra-genital *Neisseria gonorrhoeae* and *Chlamydia trachomatis* infections are mostly asymptomatic, and important reservoir sites of infection as they often go undetected and may be more difficult to eradicate with recommended therapeutic regimens. Commercial nucleic acid amplification tests (NAATs) have not received regulatory approval for the detection of *N. gonorrhoeae* and *C. trachomatis* in extra-genital specimens. The HOLOGIC® APTIMA Combo2 assay for *N. gonorrhoeae* and *C. trachomatis* has performed well in evaluations using extra-genital specimens.

**Methods:**

We assessed the performance of an in-house real-time duplex PCR assay for the detection of *N. gonorrhoeae* and *C. trachomatis* in urine and extra-genital specimens using the HOLOGIC® APTIMA assays as gold standard comparators. Urine, oropharyngeal and ano-rectal specimens were collected from each of 200 men-who-have-sex-with-men (MSM) between December 2011 and July 2012.

**Results:**

For *N. gonorrhoeae* detection, the in-house PCR assay showed 98.5–100% correlation agreement with the APTIMA assays, depending on specimen type. Sensitivity for *N. gonorrhoeae* detection was 82.4% for ano-rectal specimens, 83.3% for oropharyngeal specimens, and 85.7% for urine; and specificity was 100% with all specimen types. The positive predictive value (PPV) for *N. gonorrhoeae* detection was 100% and the negative predictive value (NPV) varied with sample type, ranging from 98.5–99.5%. For *C. trachomatis* detection, correlation between the assays was 100% for all specimen types. The sensitivity, specificity, PPV and NPV of the in-house PCR assay was 100% for *C. trachomatis* detection, irrespective of specimen type.

**Conclusion:**

The in-house duplex real-time PCR assay showed acceptable performance characteristics in comparison with the APTIMA® assays for the detection of extra-genital *N. gonorrhoeae* and *C. trachomatis*.

## Background

*Chlamydia trachomatis* and *Neisseria gonorrhoeae* are among the most prevalent sexually transmitted pathogens worldwide [1, 2]. The majority of chlamydial and gonococcal infections are asymptomatic and regular screening is recommended for at-risk sexually active individuals [3–5]. Simultaneous asymptomatic infection with both gonorrhoea and chlamydia is not uncommon, particularly among men-who-have-sex-with-men (MSM) [6, 7]. Effective screening for sexually transmitted infection (STI) pathogens is important for the appropriate management of infected individuals and their partners, and prevention of further transmission [8].

Published data support the fact that commercial nucleic acid amplification tests (NAATs) are more sensitive than culture for the diagnosis of *N. gonorrhoeae* and *C. trachomatis* infections across a range of specimen types and under varying conditions [4, 5, 9]. Even though culture is still regarded as the ‘gold standard’ for detection and diagnosis of *N. gonorrhoeae* from extra-genital sites [10, 11], multiple studies have shown that it is in fact an insensitive diagnostic tool for detecting infections at such sites [7, 12]. In the past, cell culture was performed for the detection of *C. trachomatis* in both genital and extra-genital specimens [12, 13]. These culture methods are time-consuming, expensive and labour intensive procedures that require specific and careful specimen handling and transport conditions for viable organisms to reach the laboratory [3, 10, 12, 14]. This is not always feasible, especially where there are long distances between clinical and diagnostic facilities or in resource poor settings [3, 10]. The alternative is to use NAATs, which have been shown to be more sensitive and specific for the detection of *N. gonorrhoeae* and *C. trachomatis* in urine [3], penile glans [15], ano-rectal [7, 11, 12, 15, 16] and oropharyngeal [7, 11, 12, 17] specimens. NAAT specificity varies by assay used and by anatomical site screened [7, 12, 15, 18, 19].

Regulatory approval from the US Food and Drug Administration (FDA) has been received for use of the following commercial NAATs for the detection of *N. gonorrhoeae* and *C. trachomatis* in urogenital specimens: Abbott RealTime m2000 CT/NG (Abbott Molecular Inc. Des Plaines, IL, USA), Amplicor and Cobas CT/NG test (Roche Molecular Diagnostics, Branchburg, NJ, USA), APTIMA (HOLOGIC®, San Diego, CA, USA), BD ProbeTec ET and Qx (Becton Dickinson, Sparks, MD, USA) and Xpert® CT/ NG Assay (Cepheid, Sunnyvale, CA, USA) [2]. Extra-genital pharyngeal and ano-rectal swab specimens are not officially approved for use with the APTIMA Combo 2 assay, even though they have displayed acceptable performance characteristics in evaluations [2, 9, 15, 16]. Each laboratory is required to perform their own validation and verification of data to support the results obtained from these sample sites according to the Clinical Laboratory Improvement Amendments (CLIA) regulatory requirements [15, 20].

A validated in-house real-time multiplex PCR assay is in use for the detection of *N. gonorrhoeae*, *C. trachomatis, Trichomonas vaginalis* and *Mycoplasma genitalium* infection in endocervical, vaginal, urethral swabs and male first-pass urine at the Centre for HIV and STIs (CHIVSTI), National institute for Communicable Diseases (NICD) in Johannesburg, South Africa (data not presented). However, this assay has never been evaluated for use with either oropharyngeal or ano-rectal swabs specimens.

For the purpose of this study, the validated in-house real-time multiplex PCR was modified to a duplex PCR by incorporating only the primers and probes for the *N. gonorrhoeae* and *C. trachomatis* targets. We evaluated the performance characteristics of the DNA-based in-house duplex PCR assay in comparison to the rRNA-based HOLOGIC® APTIMA assays (APTIMA Combo 2 or APTIMA GC +/− APTIMA CT) for the detection of *N. gonorrhoeae* and *C. trachomatis* in urine and extra-genital specimens.

## Methods

### Study population

This study utilised specimens that were collected during a cross-sectional study of symptomatic and asymptomatic STIs among MSM in Cape Town, South Africa [21]. MSM patients attending the Ivan Toms Centre for Men’s Health were recruited into the original study and written informed consent obtained from all participants. This is a state-sector clinic that delivers locally appropriate sexual health care such as STI and HIV screening and treatment and pre-exposure prophylaxis (PrEP) provision to gay, bisexual and other MSM, as well as the transgender community of the Western Cape, regardless of socio-economic status. A total of 200 consecutive MSM, 18 years of age or above, and reporting sexual activity with another man within the last 12 months, were enrolled from December 2011 through to July 2012. Ethics approval (Protocol number: HSREC 419/2011) was obtained from the Human Sciences Research Ethics Committee at the University of Cape Town.

### Specimen collection and transport

Ano-rectal and oropharyngeal swabs (clinician collected) and a self-collected first-pass urine (20 ml) specimen were obtained from each of the 200 participants. The oropharyngeal and ano-rectal swab specimens were collected using the APTIMA Unisex Swab Collection kit (HOLOGIC®, San Diego, CA, USA). A two millilitre aliquot of urine from each patient was transferred to an APTIMA Urine Collection kit for Male and Female specimens tube (HOLOGIC®) within 24 h after collection as per manufacturer’s instructions. All specimens were stored at 2–8 °C and transported to the laboratory where they were stored at 2–8 °C and tested within 10 days of receiving.

### Molecular testing

#### HOLOGIC® APTIMA assays

The APTIMA Combo 2 assay is a second generation NAAT probe test that uses target capture, transcription mediated amplification and dual kinetic assay technology to differentiate between the 23S rRNA of *N. gonorrhoeae* and 16S rRNA of *C. trachomatis* in clinical specimens. Assay test results, based on chemiluminescent detection, are automatically interpreted by the APTIMA assay software. Testing of the three specimen types (urine, oropharyngeal and ano-rectal swabs) was performed, according to the manufacturer’s instructions, using a 400 μl volume of each specimen. Indeterminate results on the APTIMA Combo 2 assay, or discordant results between the APTIMA Combo 2 and in-house real-time PCR assays, were resolved by using a third “tie-breaker” assay, either APTIMA GC or APTIMA CT assay, which uses different 16S rRNA primer targets from the APTIMA Combo 2 assay. The kits contain standardised positive controls for each target.

#### In-house real-time duplex PCR assay for the detection of N. gonorrhoeae and C. trachomatis infection

Genomic DNA was extracted from 200 μl urine and swab specimens stored in HOLOGIC® APTIMA collection tubes using the MagNA Pure LC DNA isolation kit I (Roche Molecular Diagnostics, Mannheim, Germany) on the automated MagnaPure LC2.0 platform (Roche). The extracted DNA was subsequently used in downstream PCR assay applications.

The in-house real-time duplex PCR assay primers and probes target the *N. gonorrhoeae* cytosine-specific DNA methyl transferase gene and the cryptic plasmid of *C. trachomatis.* For *N. gonorrhoeae* the forward primer sequence is 5′-GGA TAC GAC GTA ACC TTG ACT ATG G-3′, the reverse primer sequence is 5’-CCG ATG TAG AAG ACC CTT TTG C-3′ and the TaqMan probe sequence is 5′-[AMINO C6 + ROX] CA ACG CCA AAG ACT ACG GTG TAG CAC AG [BHQ2a]-3′. For *C. trachomatis* the forward primer sequence is 5′-GGA TTG ACT CCG ACA ACG TAT TC-3′, the reverse primer sequence is 5’-ATC ATT GCC ATT AGA AAG GGC ATT-3′ and the TaqMan probe sequence is 5′-[6-FAM]TT ACG TGT AGG CGG TTT AGA AAG CGG [BHQ1a]-3′. The 25 μl PCR master mix consisted of 5 U AmpliTaq Gold with GeneAmp 1X PCR buffer, 25 mM MgCl_2_ (Applied Biosystems, New Jersey, USA) and 400 μM dUTP mix (Bioline GmbH, Berlin, Germany). Target-specific, custom designed *C. trachomatis* primers (300 nM) and probe (200 nM) and *N. gonorrhoeae* primers and probe (200 nM each) were added to the master mix. A total of 25 μl extracted DNA was added to the master mix and the real-time PCR assay was performed on the RotorGene™ 3000/6000 (QIAGEN®) platforms as described previously [22]. DNA extracts from the *N. gonorrhoeae* ATCC strain 700,825 and *C. trachomatis* ATCC strain VR-885 were used as positive controls.

### Data analysis

The performance characteristics of the in-house real-time duplex PCR assay i.e. sensitivity, specificity, PPV and NPV for detection of *N. gonorrhoeae* and *C. trachomatis* in extra-genital and urine specimens were calculated by comparing results of in-house real-time duplex PCR and APTIMA assays. The APTIMA Combo 2 assay was considered the gold standard diagnostic method, but the APTIMA GC and/or APTIMA CT assays were used for the resolution of equivocal or discordant results with the APTIMA Combo 2 assay, in order to get a definitive end result. The percentage correlation or agreement between the assays was also calculated.

## Results

*Urine specimens:* Of the 200 urine specimens, six (3.0%) tested positive for *N. gonorrhoeae* and seven (3.5%) for *C. trachomatis* with both NAAT assays. There were no mixed infections detected in the urine specimens. One (0.5%) specimen tested positive for *N. gonorrhoeae* on the APTIMA Combo 2 assay (and was confirmed positive by APTIMA GC assay), but tested negative with the in-house duplex PCR assay.

Five (2.5%) specimens with equivocal results for *N. gonorrhoeae* on APTIMA Combo 2 assay were tested using the APTIMA GC assay and negative results, which correlated with the in-house real-time duplex PCR assay results, were obtained.

*Oropharyngeal specimens:* Of the 200 specimens, 194 specimens contained sufficient DNA extract for use in further testing. A total of 10/194 (5.2%) oropharyngeal specimens tested positive for *N. gonorrhoeae* with both NAATs. Discordant results for *N. gonorrhoeae* were observed for two specimens (1.0%) that tested negative with the in-house duplex PCR assay, but positive with APTIMA Combo 2 as well as the APTIMA GC assays. Five (2.6%) specimens with equivocal results for *N. gonorrhoeae* on APTIMA Combo 2 assay were tested using the APTIMA GC assay. The negative results obtained with the APTIMA GC assay for all five specimens correlated with the in-house real-time duplex PCR assay results. *C. trachomatis* was not detected in any oropharyngeal specimens when tested with either NAAT platform.

*Ano-rectal specimens:* Of the original 200 specimens, 199 were available for further testing. *N. gonorrhoeae* and *C. trachomatis* co-infections were detected in seven (3.5%) specimens with both assays. For *N. gonorrhoeae*, single infections were detected in 7/199 (3.5%) of specimens with both NAATs. Another three (1.5%) specimens tested positive for *N. gonorrhoeae* with the APTIMA Combo2 and APTIMA GC assays, but negative with the in-house duplex PCR assay. Six (3%) specimens resulted in APTIMA Combo 2 equivocal results for *N. gonorrhoeae* and were repeat tested with the APTIMA GC assay. All six specimens tested negative with the APTIMA GC assay, and these results were in agreement with those obtained with the in-house real-time duplex PCR assay. For *C. trachomatis*, single infections were detected in 8/199 (4.0%) specimens by both NAATs.

### Performance characteristics of the in-house real-time duplex PCR assay compared to the gold standard APTIMA assays

The performance characteristics (sensitivity, specificity, PPV and NPV) of the in-house real-time duplex PCR assay was evaluated in comparison to the gold standard APTIMA Combo2 assay, (and the APTIMA CT and/or APTIMA GC assays if an equivocal result was obtained with the APTIMA Combo2), for the detection of *N. gonorrhoeae* and *C. trachomatis* in the different specimen types tested (Tables 1 and 2). The percentage correlation between the two test methods for the detection of *N. gonorrhoeae* was 98.5% with ano-rectal specimens, 99% with oropharyngeal specimens and 99.5% with urine. For *C. trachomatis* the percentage correlation between the assays was 100% across all the specimen types tested.

**Table 1:** Performance characteristics of the in-house real-time duplex PCR assay for the detection of *N. gonorrhoeae*

Specimen type	COMPARATOR – HOLOGIC® APTIMA assays								
	Oropharyngeal swab			Ano-rectal swab			Urine		
TEST: PCR^a^	Positive	Negative	Total	Positive	Negative	Total	Positive	Negative	Total
Positive	10	0	10	14	0	14	6	0	6
Negative	2	182	184	3	182	185	1	193	194
Total	12	182	194	17	182	199	7	193	200
Sensitivity	83%			82.4%			85.7%		
Specificity	100%			100%			100%		
PPV	100%			100%			100%		
NPV	98.9%			98.4%			99.5%		
% Agreement	99%			98.5%			99.5%		

^a^PCR = in-house duplex real time PCR assay

**Table 2:** Performance characteristics of the in-house real-time duplex PCR assay for the detection of *C. trachomatis*

Specimen type	COMPARATOR – HOLOGIC® APTIMA assays								
	Oropharyngeal swab			Ano-rectal swab			Urine		
TEST: PCR*	Positive	Negative	Total	Positive	Negative	Total	Positive	Negative	Total
Positive	0	0	0	15	0	15	7	0	7
Negative	0	194	194	0	184	184	0	193	193
Total	0	194	194	15	184	199	7	193	200
Sensitivity	N/A**			100%			100%		
Specificity	100%			100%			100%		
PPV	N/A**			100%			100%		
NPV	100%			100%			100%		
% Agreement	100%			100%			100%		

^*^PCR = in-house duplex real-time PCR assay

^**^N/A = Not applicable, since no *C. trachomatis* were detected, these values could not be calculated

## Discussion

We compared the performance characteristics of an in-house real-time duplex PCR assay with that of commercial HOLOGIC® APTIMA assays for the detection of *N. gonorrhoeae* and *C. trachomatis* in urine and extra-genital specimens. The percentage correlation between the assays for the detection of *N. gonorrhoeae* ranged from 98.5–99.5% depending on specimen type tested, but for *C. trachomatis*, it was 100% for all the specimen types tested.

Even though the analytical sensitivity of the APTIMA system is unknown, it may be as low as 1 inclusion forming unit in the case of *C. trachomatis* and less than 50 cells per assay for *N. gonorrhoeae*, according to the manufacturer’s package insert. The detection limit for the in-house real-time duplex PCR assay in our laboratory is also unknown, but an evaluation conducted at the US Centres for Disease Control and Prevention (CDC) showed similar analytical sensitivity of 1–10 genomic copies of *N. gonorrhoeae* and 0.1 inclusion-forming unit of *C. trachomatis* (personal communication, Dr. Chen, CDC).

There may be several reasons for the discordant results between the APTIMA and in-house duplex PCR assays. Nucleic acid extraction was done on 200 μl of the specimen-containing transport medium tube for the in-house duplex PCR assay, whereas the APTIMA assay systems require double that volume, i.e. a minimum testing volume of 400 μl. Because of the target mediated amplification (TMA) step in the APTIMA Combo 2 assay, it can detect even a very low target copy number and increase the target sites exponentially and faster than a conventional PCR assay approach, resulting in higher amplification levels, and therefore better detection. The sensitivity is increased by the target capture of rRNA genes that are present in higher copy numbers in the bacterial cell than the target analytes of the in-house duplex PCR assay. Ideally, first void specimens should be used to increase the sensitivity of NAAT, but this was not possible for this evaluation. These factors could explain why two oropharyngeal, three ano-rectal specimens, and one urine specimen tested positive for *N. gonorrhoeae* with the APTIMA Combo 2 assay, but negative on the in-house duplex PCR assay platform. Due to the target-specific capture step during the TMA part of the APTIMA assay system most, if not all, inhibitors are removed from the system and the pure target extracted before amplification ensues. This is not necessarily the case for the non-target-specific magnetic-based nucleic acid extraction method, where all nucleic acid material from a specimen is extracted. TMA has been shown to be more sensitive than other NAATS in previous evaluations [5, 12, 15]. Another explanation for discrepancies could be that at the time of PCR testing, the duplex PCR assay did not include a target for an internal human DNA control in order to validate the quality of the extracted DNA. It is possible that DNA extraction and subsequent downstream amplification methods failed for these specimens. The issue, of including an internal human DNA control target into the in-house real-time duplex PCR assay, has since been addressed and the assay has been re-validated.

Use of the in-house real-time duplex PCR assay has certain advantages over the APTIMA Combo 2. The cost of the commercial APTIMA assay, inclusive of materials and reagents, is more than twice that of our in-house PCR assay. The duplex PCR assay can also be modified to a multiplex PCR format to additionally detect other sexually transmitted pathogens associated with urogenital discharge, such as *T. vaginalis* and *M. genitalium.* A human-DNA internal control target provides assurance that nucleic acid extracted is of sufficient quality for downstream applications. Disadvantages of the in-house real-time duplex PCR assay include the requirement for more hands-on time and training and that a maximum of only 36 specimens, inclusive of quality controls, can be tested in a single run lasting two hours. With the fully-automated APTIMA system significantly less hands-on preparation time is required for sample processing and in-process interaction. It is a random access system and the first five results will be available within three and a half hours. The results of a full run of 100 specimens, inclusive of quality controls, will be available within five hours.

There are concerns regarding the specificity of NAATs when testing samples from sites colonised by other commensal *Neisseria* species [23]. These species are genetically closely related, and may result in false positive *N. gonorrhoeae* results [9, 14, 23]. The same has not been observed with the APTIMA Combo 2 or the APTIMA GC assays [9, 17, 19]. Studies have revealed sensitivities of 100% and specificities of 99.2–99.5% for *C. trachomatis* detection in oropharyngeal swabs with APTIMA assays, compared to the BD ProbeTec ET system, which is based on strand displacement DNA amplification technology. Similarly, diagnostic sensitivity for *C. trachomatis* detection in ano-rectal swabs was reported to be 100% and specificity ranged from 98.7 to 100%. For *N. gonorrhoeae* detection in oropharyngeal swabs and rectal swabs, sensitivity was reported to be 95 and 100%, respectively; and specificities ranged from 99.6–100% and 99.5–100%, respectively [9, 12]. Therefore, APTIMA assays were deemed appropriate for use as the gold standard comparators in our assay evaluation study.

Individuals at risk of contracting STIs include key-populations such as MSM, adolescents, women ≤24 years of age and pregnant women attending antenatal clinics, that have documented high prevalence of *N. gonorrhoeae* and *C. trachomatis* infections [2, 4, 5, 21, 24]. In MSM, STIs occur at both urethral and non-urethral sites, yet screening of non-urethral or extra-genital sites such as the ano-rectum and oropharynx remains uncommon [7, 8, 11, 16, 25]. Most extra-genital infections are asymptomatic [10, 26, 27] and thus are not diagnosed using the syndromic management approach, which relies on the presence of symptoms and/or signs as the entry point to appropriate management algorithms. The most common site of asymptomatic gonococcal infection in MSM is the oropharynx [21, 25, 26], indicating that this could be an important reservoir for infection at genital sites [25]. Having the ability to screen for gonorrhoea and chlamydia at extra-genital sites with inexpensive laboratory or point-of-care tests would revolutionize STI screening in key populations, such as MSM and sex workers, in resource-poor settings.

In addition to MSM, some of whom reside within high-risk core groups or who may act as a bridge for bacterial STI transmission to the wider community [28], there is a large heterosexual population at risk of infection at extra-genital sites [16, 29]. Treatment failures in patients with *N. gonorrhoeae* infection due to antimicrobial resistance or reduced antimicrobial susceptibility are well documented [23, 30–32]. *N. gonorrhoeae* infection of the oropharynx is more difficult to eradicate than infections at urogenital sites and may therefore persist despite the administration of recommended treatment. The oropharynx is an anatomical site that facilitates the acquisition of antimicrobial resistance determinants through genetic exchange between oral commensal and pathogenic bacterial pathogens [23, 30, 32]. Additionally, extended-spectrum cephalosporins such as cefixime and ceftriaxone, recommended in current treatment regimens, may not reach adequate and consistent concentration levels in oropharyngeal tissue [30]. This can lead to selection and spread of gonococci with decreased susceptibility to antimicrobials over time, resulting in treatment failure [33]. It is therefore important to test extra-genital specimens for *N. gonorrhoeae* and *C. trachomatis* infection from at-risk individuals, using a sensitive and specific assay [11, 27, 34].

## Conclusion

Our study demonstrated that the in-house real-time duplex PCR assay showed acceptable performance characteristics in comparison to the HOLOGIC® APTIMA assays for the detection of *N. gonorrhoeae* and *C. trachomatis* from urine, oropharyngeal and rectal specimens. It would be a relatively inexpensive screening test for these genital and extra-genital infections.

## Abbreviations

CDC: US Centres for Disease Control and Prevention
CHIVSTI: Centre for HIV and STIs
CLIA: Clinical Laboratory Improvement Amendments
FDA: US Food and Drug Administration
MSM: Men-who-have-sex-with-men
NAAT: Nucleic acid amplification test
NHLS: National Health Laboratory Service
NICD: National Institute for Communicable Diseases
NPV: Negative predictive value
PCR: Polymerase chain reaction
PEPFAR: US President’s Emergency Plan for AIDS Relief
PPV: Positive predictive value
PrEP: Pre-exposure Prophylaxis
STI: Sexually transmitted infection
TMA: Target mediated amplification
